# Ultrasonication of Micellar Casein Concentrate to Reduce Viscosity—Role of Undissolved Material

**DOI:** 10.3390/foods12244519

**Published:** 2023-12-18

**Authors:** Frank Schulnies, Lisa Höhme, Thomas Kleinschmidt

**Affiliations:** Department of Applied Biosciences and Process Engineering, Anhalt University of Applied Sciences, 06366 Köthen, Germany; lisa.hoehme@hs-anhalt.de (L.H.); thomas.kleinschmidt@hs-anhalt.de (T.K.)

**Keywords:** micellar casein concentrate, sonication, hydration, voluminosity, viscosity, solubility

## Abstract

This research reveals the underlying mechanisms that make high-intensity ultrasound an effective tool to reduce the viscosity of micellar casein concentrates and to enhance the solubility of the respective powders. Micellar casein concentrates (MCC) gained great importance in the production of valuable food products with high protein content, but the processing properties of the reconstituted solutions are deficient. Even though several presumptions were established, the reasons why ultrasound is able to reduce the product viscosity and what limitations occur when using sonication technology are still not clear yet. Our study aims to investigate those reasons by combining analyses of viscosity measurements, particle size distributions, solubility, and hydration. The data presented demonstrate that undissolved, highly hydrated particles play an important role in micellar casein concentrates showing a high viscosity. We conclude on the high voluminosity of those particles, since improved solubility and decreased viscosity are accompanying effects. The determined voluminosities of those particles are 35–40% higher than for colloidal dissolved micelles. Hence, the viscosity reduction of up to 50% can be only obtained by sonicating micellar casein concentrates derived from powder reconstitution, whereas ultrasonication of freshly prepared membrane-filtrated MCC does not reduce viscosity.

## 1. Introduction

Membrane-concentrated casein products like milk protein concentrates (MPC) and micellar casein concentrates (MCC) are of major importance in the manufacture of a wide variety of food products due to their nutritional quality and functionality [1,2]. They are commonly produced by membrane filtration of skim milk followed by spray drying [3,4].

When dealing with casein-dominant products, some drawbacks may occur. One problem is associated with the fluid viscosity. Concentrating a micellar casein suspension increases the volume fraction and the viscosity sharply even at low dry matter content levels (<20%) [5,6,7,8]. The reason for this behavior lies in the high voluminosity of the casein micelles, which is in the range of 3.5–5.0 mL g^−1^, depending on, amongst others, temperature [8,9,10]. Above a volume fraction of 50%, the dispersed casein particles interact more strongly with each other, and overlapping of the κ-casein hairy layer occurs, resulting in a steeper increase in viscosity in this volume fraction range [6,11].

Controlling the viscosity of dairy concentrates is crucial for processability, i.e., pumping, wetting of evaporator tubes, and atomization during spray drying, as well as for maintaining physical and functional characteristics of the resulting powders [12,13,14]. For spray drying, it is a common practice to concentrate the feed as much as possible since the energy demand for spray drying is very high (≈3–5 MJ kg^−1^ powder [15,16]) in comparison to that for evaporation. However, as mentioned above, for casein-rich fluids, concentration is limited to low dry matter content levels.

Another issue with casein-dominant products is related to their rehydration characteristics. Since they are mostly used in powder form, dissolution behavior is an essential quality attribute, and rehydration should be quick and complete [17,18,19]. However, casein-rich powders show poor rehydration characteristics [3,20,21], which is particularly evident from their very long rehydration times [3,19,22]. This behavior is related to a cross-linking of casein micelles at the particle surface, resulting in a barrier to water transport and a delayed hydration of powder particles [20,23,24,25,26].

Overall, there is a need for strategies to improve processability of casein-rich products at higher dry matter content levels and to shorten rehydration times. A strategy that has been shown to be a promising technique for improving the functionality and viscosity behavior of dairy concentrates is the use of high-intensity ultrasound.

Ultrasound refers to mechanical waves in the inaudible range (>16 kHz) [27,28]. For food applications, high-intensity ultrasound (known as Power-US) with low frequencies (≈20 kHz) and powers >1 W cm^−2^ is typically used [29]. The sound waves that impinge on the medium generate an acoustic pressure that compresses and expands the fluid particles [30]. At high intensities, the expansion phase causes the vapor pressure to drop, resulting in the formation of cavitation bubbles that grow during several acoustic cycles and eventually implode during the compression phase [27,30]. This creates locally very high pressures (>1000 bar), temperatures (≈5000 K), and shear forces [31,32]. These effects of acoustic cavitation can be utilized for many food applications, including emulsification/homogenization [33], extraction processes [34], and crystallization [35], as well as filtration [36] and microbial inactivation [37].

Different reasons have been discussed for the decreased viscosity due to sonication, including improved solubility, changes in particle size, changed conformation, and the resulting higher interactions between water and protein [38].

Since acoustic cavitation can lead to disruption of polymer chains, it was assumed that it would also disrupt weakly associated proteins [39]. In contrast, other experimental setups led to the conclusion that solubility is improved due to shear forces and a decrease in particle size, but that casein micelles do not endure structural changes [40,41]. Generally, it is not proven yet which mechanisms lead to structural changes in proteins and enzymes as an especially susceptible form of protein. Effects due to formation of radicals, attacking the protein backbone, as well as strong shear forces due to cavitation and the resulting disruption, are discussed [42,43,44]. Several studies agree that radical formation is very limited at 20 kHz and therefore should not be the main reason for the changed properties [45,46]. Conformational changes are discussed, especially regarding globular proteins such as whey and several plant proteins [41,47,48]. Ultrasound-induced changes in the conformation can lead to exposed hydrophilic moieties, which, in turn, increase the interactions between water and protein and finally increase solubility [47].

Ashokkumar et al. [32] stated that the particle size distribution in whey protein concentrate changed towards smaller particles sizes due to sonication and attributed this effect to the disruption of larger aggregates since they did not determine an improved solubility. The same was assumed by Desphande and Walsh [49] for the decreased viscosity in MPC, even though they considered an increased solubility as a second reason. Those assumptions omit the fact that decreasing the particle size does not necessarily lead to reduced viscosity since the volume fraction does not change and dissolution of more casein micelles should increase the viscosity instead. Therefore, a reduction in particle size cannot explain the effects, except that it is accompanied by a reduced voluminosity.

The study presented within this article aims to ascertain why sonication leads to decreased viscosity and which mechanisms are underlying. Hence, in addition to viscosity, the dissolved amount and the particle size distribution are analyzed. Based on the results, the findings are further elaborated by evaluating the hydration as well as comparing between reconstituted and membrane-filtrated MCC.

## 2. Materials and Methods

### 2.1. Preparation of Micellar Casein Suspensions

Commercial micellar casein concentrate powder (Refit^®^ by Friesland Campina (Amersfoort, The Netherlands) friendly donated by Milchwerke Mittelelbe GmbH, Stendal, Germany) was used to prepare MCC suspensions. The chemical composition of the powder is given in Table 1. A series of trials was carried out with a powder concentration of 20% (*w*/*w*) in ultrafiltration permeate, which was obtained by ultrafiltration (Pall Centramate^TM^, Pall GmbH, Bad Kreuznach, Germany) of reconstituted skim milk (10% *w*/*w*, rehydrated for 24 h).

The MCC was mixed with the permeate using a magnetic stirrer (350 rpm) for 5 h at 55 °C. The mixture (300 g total) was then stored overnight at room temperature for further rehydration. Sodium azide (purchased from Roth with purity >99%) was added to prevent microbiological growth. The dry matter content of the rehydrated MCC suspensions was determined by oven drying (105 °C, 24 h).

### 2.2. Ultrasonication of MCC Suspensions

Ultrasonication of MCC suspensions was carried out using a 20 kHz ultrasonic device (UIP 1500hdT, Hielscher Ultrasonics GmbH, Teltow, Germany) equipped with a Booster B2-1.8 and a 34 mm diameter sonotrode BS4d34. Ultrasonication was performed at an amplitude of 80% (34 µm).

**Figure 1:**
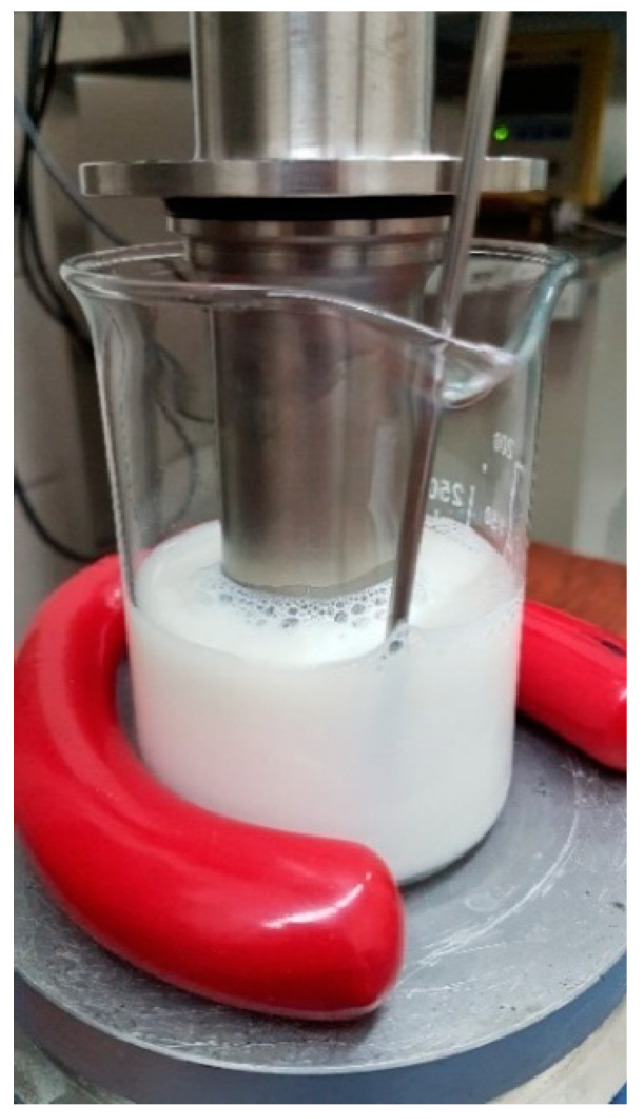
Batch ultrasonication of micellar casein suspensions.

A 125 g amount of the reconstituted MCC suspensions was transferred to a 250 mL beaker, which was placed centrally under the sonotrode on a magnetic stirrer plate. The sonotrode was immersed 1.5 cm into the solution, and the magnetic stirrer was started before sonication. Sample temperature was measured with a temperature probe connected to the sonication device. Sonication was either performed without temperature control (beaker held in air, temperature increased during sonication) or with cooling of the samples during sonication using a water bath filled with ice (trials with temperature control; sample temperature remained almost constant). The sonication duration was controlled indirectly by presetting the desired energy input, which was chosen based on preliminary experiments. After reaching the energy limit, the sonication was automatically stopped by the device. All test parameters (time, temperature, energy, amplitude, power) were recorded every second by the device. Energy density was then calculated by dividing the energy input by sample weight. Figure 1 shows an example of the experimental setup for the sonication of an MCC solution without temperature control. All trials and subsequent analyses were conducted in duplicates and compared to a sample without sonication as the reference sample.

### 2.3. Viscosity Measurements

The viscosity of the MCC suspensions was measured at 23 °C using an Anton Paar rheometer (MCR 302, Anton Paar Germany GmbH, Ostfildern-Scharnhausen, Germany) equipped with cone–plate measuring geometry. Viscosity data were collected over a logarithmic shear ramp from 1 s^−1^ to 1000 s^−1^ with a variable logarithmic measurement point duration from 12 s to 2 s. In case of a temperature increase resulting from ultrasonication, the samples were cooled to room temperature before starting the viscosity measurements.

### 2.4. Particle Size Measurements

Particle size distribution (PSD) was measured before and after sonication of the MCC suspensions using a Mastersizer S (Malvern Panalytica GmbH, Kassel, Germany). The suspension was added dropwise in deionized water to the wet dispersion unit until an obscuration of 10–15% was reached. For estimation of the size distribution, the presentation code 3PAD (refractive index of particles 1.59, refractive index of solution 1.33) was used.

The particle size was also determined with a Zetasizer Nano ZS (Zetasizer Nano ZS, Malvern Panalytica GmbH, Kassel, Germany). Therefore, MCC suspensions were diluted 1000-fold with permeate. Light scattering of the samples was measured at an angle of 173°. Size results were expressed as intensity-weighted harmonic mean particle diameter (z-average).

### 2.5. MCC Solubility and Hydration of Undissolved Material

The solubility of the MCC powder was determined by measuring the dry matter of the whole sample and the dry matter of the supernatant of this sample after centrifugation at 3800× *g* for 5 min (Centrifuge 5416, Eppendorf SE, Hamburg, Germany) according to Equation (1).

(1) $$\mathrm{dissolved}\;\mathrm{amount}\;(\%)=\frac{\mathrm{dry}\;{\mathrm{matter}}_{\mathrm{supernatant}}}{\mathrm{dry}\;\mathrm{matte}r_{\mathrm{whole}\;\mathrm{sample}}}\cdot \;(100\%)$$

For this purpose, the exact sample weight was measured, then, the sample was dried at 105 °C for 24 h in an oven, and, after cooling it down in a desiccator, the weight of the dry sample was measured. Samples with 20% dry matter, or no matter if dissolved in deionized water or permeate, were diluted to 5% before the determination to reduce the viscosity and enable proper sedimentation of undissolved material via centrifugation.

For the solubility kinetics, deionized water or permeate was preheated to 55 °C using a water bath (HBR Digital, IKA-Werke GmbH & Co. KG, Staufen, Germany), and the powder was mixed in to obtain a 10% or 20% (*w*/*w*) solution. The stirring speed of the magnetic stirrer was set to 450 rpm (200 g overall mass) or 600 rpm (400 g overall mass). Samples were taken every hour for five hours and analyzed for viscosity, dissolved amount, and PSD.

For the solubility trials of 10% MCC in deionized water, the hydration of the sediment, or rather the undissolved powder particles, was additionally analyzed using a drying oven overnight as described above and calculated according to Equation (2).

(2) $$\mathrm{Hydration}\left(\frac{g_{\mathrm{water}}}{g_{\mathrm{dry}\;\mathrm{matter}}}\right)=\frac{\left({\mathrm{weight}}_{\mathrm{sample}}\left(g\right)-{\mathrm{weight}}_{\mathrm{dried}\;\mathrm{sample}}(g)\right)}{{\mathrm{weight}}_{\mathrm{dried}\;\mathrm{sample}}(g)}$$

Furthermore, the samples were stored for 24 h at room temperature, and the measurements were repeated.

### 2.6. Production and Sonication of Membrane-Filtrated MCC

A membrane-filtrated MCC was produced by microfiltration of commercial UHT-treated skim milk at room temperature using a membrane cassette with a pore size of 0.2 µm (Pall CentramateTM, Pall GmbH, Bad Kreuznach, Germany). Three liters of skim milk was concentrated to a volume concentration ratio (VCR) of 3 followed by 4 diafiltration steps with deionized water. During diafiltration, the retentate was concentrated to a VCR of 4. The retentate was then concentrated to 20% dry matter by vacuum evaporation (Laborota 40003, Heidolph Instruments GmbH & Co. KG, Schwabach, Germany) at 100 mbar and 70 °C water bath temperature. After adding sodium azide, the concentrated MCC was stored overnight at room temperature until further analysis. Protein content of the concentrate was determined by Kjeldahl with a conversion factor of 6.38. Ultrasonication (without temperature control) and measurement of PSD and viscosity were carried out as described in the previous sections.

## 3. Results and Discussion

### 3.1. Effect of Ultrasonication on Viscosity of Reconstituted MCC Suspensions

**Figure 2:**
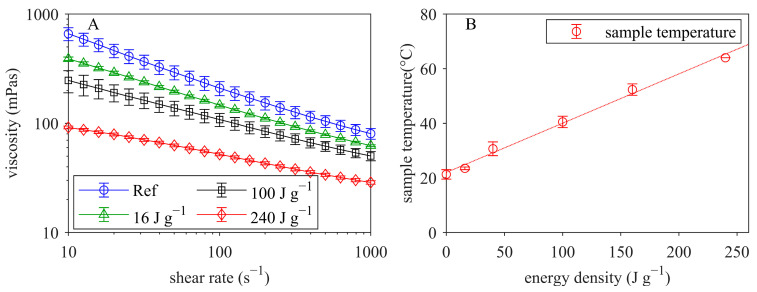
Viscosity (at 23 °C) versus shear rate (**A**) for reconstituted MCC suspensions (MCC dry matter 18.5% in permeate) that were untreated (reference, Ref) and ultrasonicated with different energy densities (without temperature control) as well as temperature development depending on energy density (**B**).

Figure 2A shows the viscosity as a function of shear rate for the reconstituted MCC suspensions (MCC dry matter 18.5% ± 0.2% in permeate) that were untreated (reference, Ref) and ultrasonicated (without temperature control) with different energy densities.

It can be seen that viscosity decreased over the entire shear rate range with increasing ultrasound energy density. All samples displayed shear thinning behavior, which decreased with increasing energy input. Ultrasonication without temperature control increased the sample temperature linearly with increasing energy density (Figure 2B). At the highest energy density of 240 J g^−1^, a sample temperature of 64 °C was reached.

Non-Newtonian behavior of casein micelle suspensions at high concentration was also observed in other studies [6,7,50]. At high volume fractions, interactions between casein micelles may lead to some kind of clustering [51]. For skim milk concentrates, it is assumed that casein micelles are weakly flocculated at high dry matter content levels [52,53]. The clusters contain trapped interstitial serum, thereby increasing the effective volume fraction, especially at low shear rates [51]. When the shear rate increases, clusters are loosened and serum is released, and thus viscosity decreases like in the case of concentrated fat globules in cream [51]. In addition, high shear rates may deform the micelles and align them in the flow direction, thereby decreasing viscosity [6].

**Figure 3:**
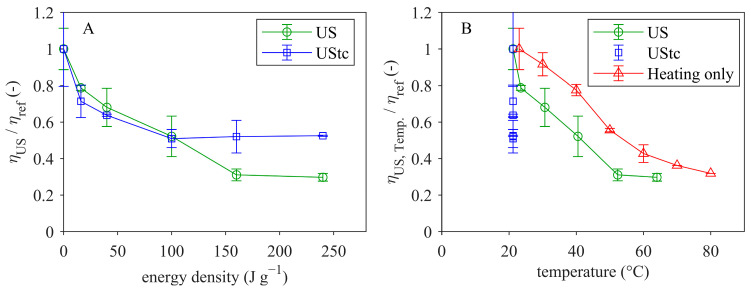
Normalized viscosity (at 23 °C) of MCC in permeate as a function of energy density (**A**) and sample temperature (**B**).

For a better comparison of the effects of ultrasonication, viscosity values of the sonicated samples at 100 s^−1^ were used and normalized to the untreated sample (185 ± 21 mPas). Normalized viscosity (η_US_/η_ref_) is shown in Figure 3A as a function of energy density for the sonication trials without (US) and with temperature control (UStc). Viscosity decreased already at low energy densities of 16 J g^−1^ by about 20–30%. Up to an energy density of 100 J g^−1^, viscosity decreased in the same manner for both treatments. However, with further increase in energy, the viscosity reduction kept constant at 50% for the sonicated samples without temperature increase. In contrast, sonication with accompanying temperature increase resulted in a higher viscosity reduction of up to 70%. Thus, it seems that higher temperatures have an additional viscosity-reducing effect during ultrasonication. In order to determine the effect of the temperature only (without ultrasound), samples were heated to different temperatures between 30 °C and 80 °C (30 s holding time) and cooled to room temperature before viscosity measurement. These results are included in Figure 3B in comparison to the sonicated samples. It is obvious that heating alone without ultrasonication caused a considerable decrease in viscosity of up to 70% with increasing sample temperature although the samples were cooled before viscosity measurement. Furthermore, as shown in Figure 3B, the viscosity decrease slowed down at higher temperatures. Ultrasonicated samples exhibited a higher viscosity reduction at comparable sample temperatures. When comparing the two ultrasound treatments, it can be seen that temperatures of above 40 °C resulted in a greater viscosity reduction.

There are different reasons for the observed viscosity reduction upon ultrasonication and heating. The temperature effect on viscosity can be described by the reduced casein micelle voluminosity since casein micelle voluminosity is known to decrease with increasing temperature [8,9,54,55]. According to Nöbel et al. [8], a temperature increase from 23 °C to 60 °C resulted in a decrease in voluminosity of 13%, from 4.0 mL g^−1^ to 3.5 mL g^−1^. This reduction in voluminosity could explain the viscosity decrease of 60% for the MCC suspensions that were heated to 60 °C without ultrasound. In addition, Nöbel et al. [55] found that the voluminosity decrease continuously slowed down with increasing temperature. This behavior was also observed for the viscosity reduction with increasing sample temperature, as shown in Figure 3B. Thus, there seemed to be a clear correlation between the voluminosity and viscosity development in MCC suspensions. However, since the samples were cooled before measurement, there was no direct temperature impact on the suspensions. Temperature kinetic studies from Liu et al. [56] with skim milk showed that re-equilibration of the casein micelle size and hydration is much slower upon cooling in comparison to the dynamic response of the mineral system. Therefore, it can be assumed that micelle voluminosity was not re-equilibrated and was still lower after temperature had been removed from the MCC suspension.

In addition to the sample temperature, which had an additional viscosity-reducing effect during ultrasonication at temperatures above 40 °C (Figure 3B), ultrasonication without a temperature increase resulted also in a significant viscosity reduction of up to 50%. The question that arises here is: what caused this marked viscosity decrease? In a study by Zisu et al. [57], membrane-concentrated MPC (18% dry matter) was ultrasonicated using a flow-through setup, which resulted in a viscosity reduction of 30%. They suggested that acoustic cavitation may break up casein–casein and/or casein–whey protein interactions. However, neither particle sizes nor temperature development of the retentate samples were reported. Li et al. [58] investigated the effect of hydrodynamic cavitation on the viscosity of nanofiltrated MPC (dry matter 23.5%) using an APV Cavitator. With this treatment, viscosities could be reduced by 20–56%. They described a structural breakdown as a reason. Particle sizes of the retentate as well as temperatures were not given. In another study, Desphande and Walsh [49] reported viscosity reductions of 27–55% for reconstituted MPC with different dry matters using batch sonication. It was assumed that the reduction in viscosity was mainly a result of breaking protein aggregates as well as an increase in the solubility of reconstituted suspensions [50]. For whey protein concentrates, different studies showed that viscosity and particle sizes are reduced after ultrasonication [57,59,60,61]. To summarize, particle size reduction and/or breakdown of aggregate structures are discussed as reasons for the observed viscosity reduction in most studies.

### 3.2. Effect of Ultrasonication on Particle Size

**Figure 4:**
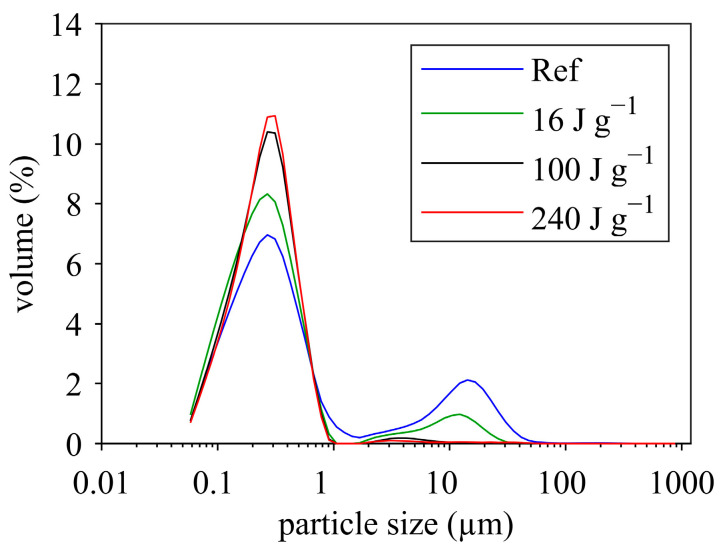
Particle size distributions of untreated and sonicated (with temperature increase) MCC suspensions (MCC dry matter 18.5%).

As reviewed in the previous section, ultrasonic treatment is effective in decreasing particle size of protein suspensions. In order to determine the extent of particle size changes in the MCC suspensions, particle size was measured before and after sonication using laser diffraction and dynamic light scattering. Figure 4 displays the particle size distribution (laser diffraction) for the untreated and sonicated samples. The untreated sample (Ref, reference) showed a bimodal size distribution with two distinct peaks at around 0.2–0.3 µm and 20 µm. The left peak represents the casein micelles, which have an average hydrodynamic diameter of ≈200 nm [62]. The right peak most probably reflects undissolved powder particles. Increasing ultrasound energy density led to a reduction in the volume of large particles and, at the same time, to an increase in the volume of casein micelles. At 100 J g^−1^ and 240 J g^−1^, almost all coarse particles disappeared. Thus, ultrasonication dissociated undissolved powder particles and released casein micelles. The same effect was observed during ultrasound-assisted dissolution of MPC powders [63].

**Figure 5:**
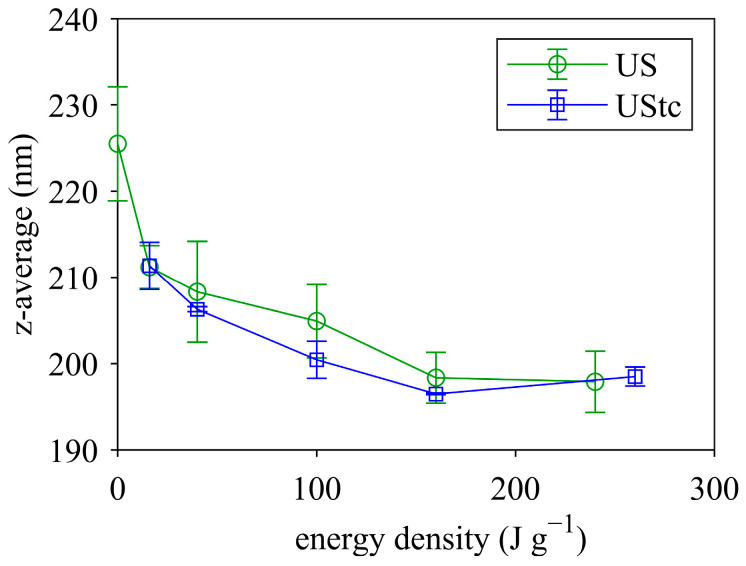
Mean particle size (z-average) as a function of ultrasound energy density for MCC suspensions (MCC dry matter 18.5%) that were ultrasonicated with (US) and without (UStc) temperature increase.

The mean particle size measured by the Zetasizer (z-average) also indicated a particle size reduction in the sonicated samples (Figure 5). No major differences were detected between ultrasonication with and without temperature increase. Increasing energy density reduced the mean particle size by up to 30 nm. It also became evident that size reduction was more pronounced below 100 J g^−1^ and slowed down at higher energy densities.

The question that arises here is: which particles were actually disrupted, casein micelles or fat droplets? The latter, although of low quantity in the MCC suspension, contribute significantly to the light scattering properties of milk [64]. In addition, fat globules were found to be of similar size to larger casein micelles (200–300 nm) [64]. Thus, the reduction of fat globule size may be responsible for the observed decrease in the average particle size, as also found by Chandrapala et al. [64] after sonication of skim milk. However, other studies reported a decrease in micelles size upon ultrasonication [65,66,67]. Therefore, some uncertainty remains as to whether or not casein micelles are disrupted during ultrasonication. Nevertheless, there was a clear reduction of undissolved coarse particles with increasing energy density (Figure 4), which could be the reason for the decrease in viscosity of the MCC suspensions upon ultrasonication.

In order to obtain a better understanding of the influence of the undissolved particles on viscosity reduction, further experiments were conducted with respect to the solubility–viscosity relationship and the hydration of the undissolved material.

### 3.3. Solubility–Viscosity Relationship of MCC Suspensions

**Figure 6:**
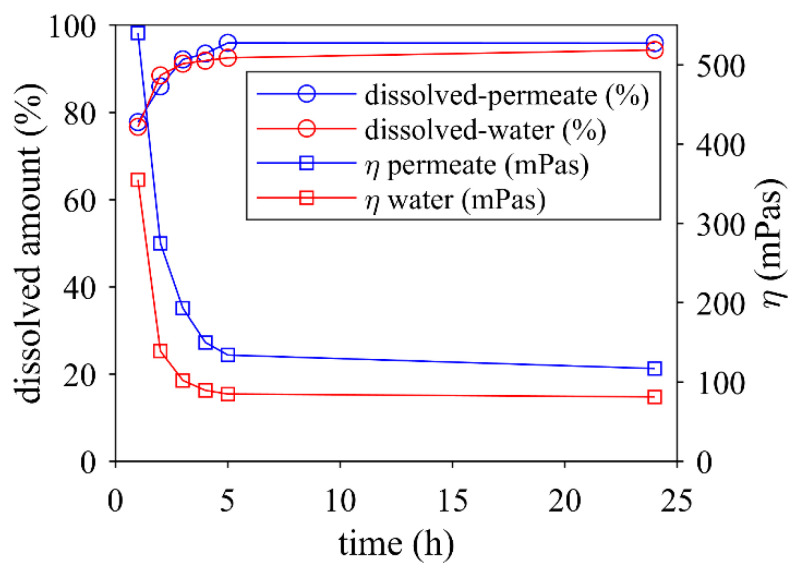
Solubility and viscosity of MCC dissolved in water (dry matter 19.4%) and permeate (dry matter 18.2%) at 55 °C (magnetic stirrer) as a function of rehydration time.

Figure 6 displays the solubility–viscosity relationship for MCC dissolved in water (dry matter 19.4%) and permeate (dry matter 18.2%). There was a significant reduction in viscosity and increase in solubility over the first 3 h, followed by a slight reduction and increase over the remaining dissolution time. The solubility in permeate and water, as well as the relative viscosity reduction during dissolving, was comparable, but the absolute viscosity of the MCC in permeate was higher. This might be due to the higher voluminosity of the micelles in the salt-containing surroundings [56].

The increase in solubility due to the dispersion of undissolved particles and release of casein micelles seem to play an important role in the viscosity reduction, as already described by other authors [22,40,49]. Interestingly, the solubility of the MCC was high (95–97%) after 24 h, and only a small amount was undissolved. Thus, it was surprising that, even at this high solubility level, viscosity could be further reduced by 50% upon ultrasonication (Figure 3A). This fact implies that small amounts of undissolved material can also have a huge influence on viscosity.

From a theoretical point of view, particle size reduction per se does not reduce effective volume fraction and thus viscosity as long as the voluminosity of the dispersed particles remains unchanged. Consequently, it can be assumed that the undissolved particles in the MCC suspensions exhibited a higher hydration and voluminosity in comparison to the colloidal dissolved casein micelles and that the disruption of the undissolved material resulted in a decrease in both parameters. The higher voluminosity of undissolved particles may result from the swelling of MCC particles during the first stage of rehydration. Gaiani et al. [22] observed an increase in particle size and viscosity during the early stage of dissolution of native phosphocaseinate. However, the extent of hydration and voluminosity increase in undissolved particles is still unknown.

### 3.4. Hydration of Undissolved Material

To obtain a better idea of the hydration of the undissolved material, solubility experiments were conducted with MCC (10% dry matter) in water. MCC in water was used for further trials since the dissolution behavior was the same no matter whether it was dissolved in water or permeate (Figure 6). This enabled an easier sample preparation and avoided effects due to minor changes in the permeate composition.

**Figure 7:**
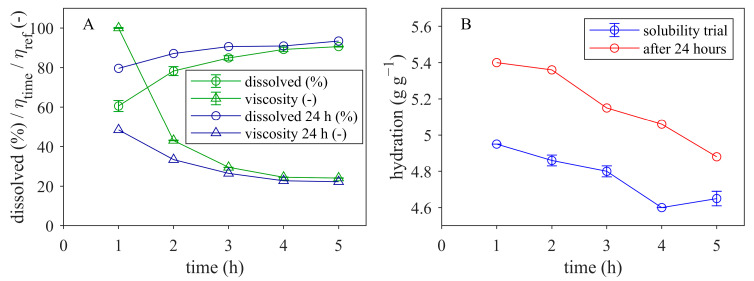
Solubility and normalized viscosity of 10% MCC in water (**A**) and hydration of sediment during the solubility trial for 5 h at 55 °C as well as after storage overnight at room temperature (**B**) as a function of dissolving time.

The hydration of the undissolved material was 4.7 g g^−1^ after 5 h at 55 °C and increased during storage overnight at 23 °C to 4.9 g g^−1^. The increase was even higher for shorter dissolution times, e.g., for 2 h at 55 °C to 4.8 g g^−1^ and after storage overnight at 23 °C to 5.4 g g^−1^, as shown in Figure 7. Hence, the particles underwent further swelling overnight.

According to Huppertz et al. [10], casein micelles exhibit a hydration of 3.3 g g^−1^. Compared to this, the undissolved particles showed a 40% higher hydration after 5 h dissolution and a 50% higher hydration after storage and concomitant swelling overnight. To calculate voluminosity, Equation (3) [68] was used, where ν is the voluminosity, δ is the hydration, and V_P_ and V_W_ are the partial specific volume of the dry protein and water, respectively.

(3) $$\nu \;\left(\frac{ml}{g_{dry\;matter}}\right)=V_{P\;}\left(\frac{ml}{g_{dry\;casein}}\right)+\delta (\frac{g_{water}}{g_{dry\;matter}})\cdot V_{w}(\frac{ml}{g_{water}})$$

Since the undissolved particles are built up by casein micelles, the specific volume of dry casein micelles (0.7 mL g^−1^ [9] (p.143)) was taken as a value for V_P_. From the hydration experiments (Figure 7B), values of 4.7 g g^−1^ and 4.9 g g^−1^ were taken as hydration for the undissolved material. This resulted in a voluminosity of 5.4 mL g^−1^ and 5.6 mL g^−1^, respectively. Thus, in comparison to a voluminosity of 4.0 mL g^−1^ for casein micelles, the undissolved particles exhibited a 35–40% higher voluminosity. Assuming that the undissolved amount is 5%, the volume fraction will thereby increase by approximately 2% absolute, starting from a solubility of 100%. This seems to be very little to explain the huge viscosity differences observed before and after ultrasonication. However, the viscosity of casein suspensions is known to increase sharply at a volume fraction >50% [6,7,11], and, thus, even small changes in volume fraction could result in a drastic increase or decrease in viscosity.

### 3.5. Comparison of the Effect of Centrifugation and Ultrasonication

High-intensity ultrasound seems to be suitable to immediately dissolve highly hydrated particles; thus, the viscosities of a sonicated sample having the same dry matter as a supernatant after centrifugation and this supernatant should be equal. To avoid effects due to the rising temperature during sonication, the supernatant from centrifugation was heated to the final temperature reached at the end of sonication, and both samples were left to cool down before the measurements. The results obtained are shown in Table 2.

**Figure 8:**
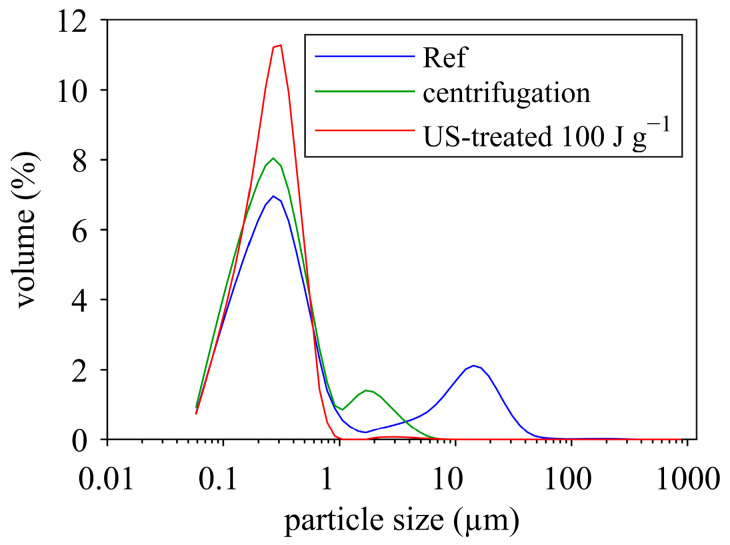
Particle size distributions of untreated (Ref), centrifuged (5 min), and sonicated MCC suspensions (MCC dry matter 10%).

As expected, centrifugation as well as sonication led to a decreased viscosity, but the decrease was far higher for the sonicated samples. Just diluting to the dry matter of the supernatant already accounted for 15% viscosity reduction, but the centrifugation and removing of undissolved particles led to a 35% reduction in viscosity. Sonication decreased the viscosity further by almost 60% compared to the reference. Comparing the three samples obtaining the same dry matter (supernatant 5 min; sample untreated, 8.9% DM; sonicated sample), sonication reduced the viscosity by 50%, whereas centrifugation only reduced viscosity by 24%. To state it in another way, the untreated sample (8.9%DM) had twice the viscosity of the sonicated sample, and the centrifuged sample still had 1.5-fold viscosity compared to the sonicated sample. The reason for this result was partly visible when using longer centrifugation times, which led to a lower dry matter content, indicating that the centrifugation parameters chosen did not sediment all undissolved material. Regarding the viscosity, there were only minor changes comparing five and ten minutes of centrifugation time, but the particle size distribution still revealed a second peak after centrifugation, which completely disappeared after sonication (see Figure 8).

Summing up the results, the remaining peak seems to present small remainders of not fully dissolved fine material (1–8 µm) and, therefore, material possessing a higher voluminosity compared to fully colloidal dissolved casein micelles. Consequently, there seemed to be highly hydrated, undissolved coarse particles, which were removed by centrifugation, but also highly hydrated “fines”, which could not be removed by centrifugation but could be destroyed by sonication. This high hydration increases the overall volume fraction and thereby hydrodynamic interactions during shear flow.

**Figure 9:**
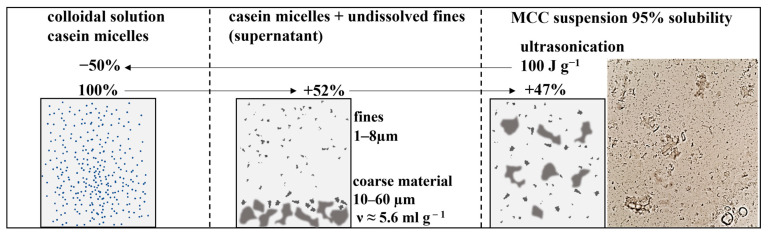
Role of undissolved material in viscosity development of MCC suspensions. Values for viscosity increase (+) and viscosity decrease (−) are normalized to the viscosity of the colloidal solution and to the viscosity of the suspension, respectively.

Overall, the role of the undissolved material in viscosity development and the effect of ultrasonication are highlighted in Figure 9. Starting from the colloidal solution, the viscosity is increased by ≈50% (at constant dry mass) due to undissolved fine particles. A further increase in viscosity of around 50% (at constant dry mass) is caused by undissolved coarse particles having an estimated voluminosity of 5.6 mL g^−1^. Ultrasonication of MCC suspensions using 100 J g^−1^ decreases viscosity by 50% due to disruption of undissolved coarse and fine material.

### 3.6. Effect of Ultrasonication on Membrane-Filtrated MCC

**Figure 10:**
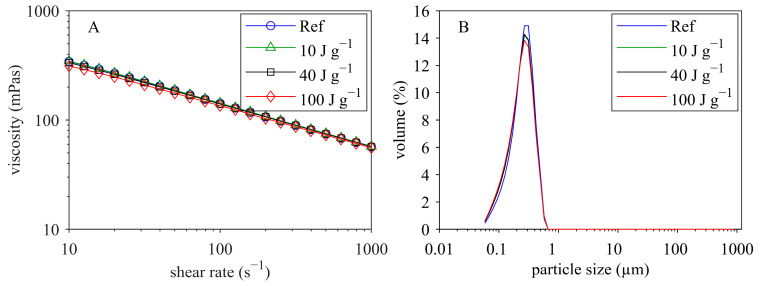
Viscosity versus shear rate (**A**) and particle size distribution (**B**) before and after ultrasonication of membrane-filtrated MCC (dry matter 19.6 ± 0.1%).

As discussed, ultrasonication of reconstituted MCC reduced viscosity due to disruption of highly hydrated, voluminous, undissolved coarse and fine material (Figure 9). Freshly prepared membrane-filtrated and concentrated MCC is a colloidal solution consisting of casein in its ‘native’ (micellar) form with no undissolved material. Therefore, sonication can cause no particle disruption, and the overall volume fraction is expected to remain constant. In addition, it is assumed that casein micelle integrity is not influenced by sonication [64]. Hence, it can be expected that sonication will have no major effect on viscosity. This assumption is supported by the results shown in Figure 10. Ultrasonication (with temperature increase) of the membrane-filtrated MCC (protein content 86% on dry basis) had neither a significant effect on viscosity (Figure 10A) nor on particle size distribution (Figure 10B). The temperature effect on viscosity, as discussed in Section 3.1, was eliminated by heating the samples to 55 °C (and cooling) before sonication. Thus, ultrasound alone was not able to reduce viscosity when the samples were in a complete colloidal state.

## 4. Conclusions

In this study, reconstituted and membrane-filtrated MCC was ultrasonicated to reduce viscosity. For reconstituted MCC, a viscosity reduction of 50% was achieved at both low and high suspension dry matter levels. However, ultrasonication of freshly prepared membrane-filtrated MCC had no effect on viscosity. It was found that the disruption of undissolved coarse and fine material was responsible for the viscosity decrease, although the undissolved amount was low for the original suspensions. Thus, the hydration and voluminosity of the undissolved material must have been high to account for the viscosity development.

Further experiments revealed that the hydration of the undissolved coarse material was 40–50% higher than the hydration of casein micelles, suggesting that even small amounts of undissolved particles are able to increase viscosity to a great extent. In addition, centrifugation experiments showed that undissolved fines remained in the supernatant of the original suspension, causing a 50% higher viscosity in comparison to the sonicated sample with the same dry matter as the supernatant. Therefore, the undissolved fine material seemed to have the same significance for viscosity development in MCC as the undissolved coarse material.

The findings presented within this study emphasize the importance of the dissolving step for industrial processes based on protein suspensions. The issues of undissolved particles such as lumping, blockage of tubing, and low stability are already well known, and the results reveal that high viscosity is also intensified by insufficient dissolved particles. The difficulty is that already small amounts of highly hydrated “fines” can increase the viscosity by 50%. Even in a solution that seems to be well dissolved, with a determined solubility of 95%, the combination of the highly hydrated coarse and fine material can double the viscosity. Therefore, ultrasound or a technique offering comparable shear effects is a promising tool to overcome this issue and improve processes where high viscosity is the limiting factor for capacity and processing parameters.

## Figures and Tables

**Table 1:** Chemical composition of micellar casein concentrate (MCC) used in this study.

Component	% Dry Basis
Protein	88.0
Casein	82.7
Lactose	3.0
Fat	1.2
Ash	7.7
Moisture	4.8

**Table 2:** Comparison of dry matter (DM), viscosity, and relative viscosity before (untreated) and after centrifugation and sonication.

	Untreated	Supernatant Centrifugation		Suspension with Same DM as Supernatant 5 min	
	Reference	5 min	10 min	Untreated	Sonicated
dry matter (%)	9.44	8.73	8.34	8.87	8.73
η @ 100 s^−1^, 23 °C (mPas)	7.42	4.82	4.71	6.31	3.16
η/η_untreated, 9.4% DM_ (%)	100	65	63	85	43
η/η_untreated, 8.9% DM_ (%)	-	76	-	100	50
η/η_sonicated_ (%)	-	152	-	200	100

## Data Availability

The data used to support the findings of this study can be made available by the corresponding author upon request.
